# Soil phosphorus fractionations as affected by cropping systems in the central mid-hills region of Nepal

**DOI:** 10.1371/journal.pone.0307139

**Published:** 2024-09-24

**Authors:** Dinesh Khadka, Keshab Raj Pande, Bhaba Prasad Tripathi, Roshan Man Bajracharya

**Affiliations:** 1 Department of Soil Science and Agricultural Engineering, Agriculture and Forestry University, Rampur, Chitwan, Nepal; 2 National Soil Science Research Centre, Nepal Agricultural Research Council, Khumaltar, Lalitpur, Nepal; 3 Department of Soil Science and Agri-Engineering, Institute of Agriculture and Animal Science, Tribhuvan University, Kirtipur, Kathmandu, Nepal; 4 Department of Environmental Science and Engineering, Kathmandu University, Dhulikhel, Kavre, Nepal; ICAR-Indian Institute of Soil Science, INDIA

## Abstract

Soil plays a critical role as the primary reservoir of phosphorus (P) in terrestrial ecosystems. Sequential fractionation has been extensively utilized to gain insights into the characteristics and dynamics of soil P. However, there is a knowledge gap regarding the different P pools in Nepalese soils. Therefore, this study aimed to investigate the impact of cropping systems on soil P fractions in the central mid-hills of Nepal. The study focused on four cropping systems: vegetable, fruit, rice, and maize-based systems, which exhibited variations in nutrient management, topography, and cropping intensity. A total of 240 soil samples (60 samples from each cropping system) were collected from multiple sites within the central mid-hill region. Standard analytical methods were used to determine the general parameters of the soils, while the sequential fractionation method was employed to assess the organic and inorganic P pools. The results indicated that the effect of cropping systems on soil pH, calcium carbonate (CaCO_3_) content, and the proportion of sand, silt, and clay was not statistically significant in terms of general parameters. However, significant differences were observed among the different cropping systems in organic matter (OM), electrical conductivity (EC), cation exchange capacity (CEC), and available phosphorus. Similarly, in terms of inorganic phosphorus fractions, loosely bound P (LB-P), aluminum bound P (Al-P), iron bound P (Fe-P), and reductant soluble P (RS-P) were significantly affected, while calcium bound P (Ca-P) did not show a significant difference. Furthermore, in terms of organic phosphorus fractions, labile organic P (L-Po), fluvic acid organic P (FA-Po), and non-labile organic P (NL-Po) exhibited significant differences, whereas moderately labile organic P (ML-Po) and humic acid organic P (HA-Po) did not show a significant difference. Additionally, reductant soluble P showed a significant difference, while total P did not differ significantly. The vegetable-based system exhibited higher levels of the majority of P fractions, followed by the fruit-based, maize-based, and rice-based systems. These findings emphasize the importance of considering cropping systems and their response to different phosphorus pools, as this knowledge can contribute to the development of improved soil phosphorus management strategies and promote sustainable agricultural practices in the region.

## Introduction

Nepal is a land-locked, Himalayan country with China (Tibet) to the north and India on its southern border. Agriculture, principal economic activity of Nepal, provides 24.90% of GDP and employing about 60.40% of the population [1]. Agriculture lands accounts for about one-third of total land resources available in Nepal [2]. Geographically, the country is divided into three ecological regions mainly running from east to west: high-hills, mid-hills, and terai accommodating 35%, 42%, and 23**%** of the total area, respectively [3]. Moreover, central Nepal belongs to all the parts of Bagmati province and most of the parts of Madhesh province (Parsa to Dhanusha). The different cropping systems such as vegetable-based, fruit-based, rice-based, maize-based, etc. are common in the central mid-hill region of Nepal.

Next to nitrogen, P is considered a major limiting nutrient for food production globally [4]. Soil phosphorus is a crucial macronutrient that restricts agricultural productivity in numerous regions of Nepal [5]. P is a major element required by plant for their growth and development. Therefore, maintaining adequate amount of soil P is critical for the long-term sustainability of cropping system [6]. The available parent material as well as inorganic and organic nutrient source applied in the field, are considered sources of P in the soils [7].

In Nepal, P is the second most nutrient applied externally, primarily in the form of diammonium phosphate (DAP) and single super phosphate (SSP), after nitrogen (N) applied in the form of urea [8]. Nepal does not have its own fertilizer production plant, hence demands are met through fertilizer imports. Due to inadequate availability and price fluctuations, the sales of DAP have been decreasing over the past three years, with quantities of 160,756.50, 140,166.63 and 77,719.87 Mt imported in 2019/20, 2020/21 and 2021/22, respectively [2]. However, in areas where organic sources of nutrients are insufficient, the application of inorganic fertilizer such as DAP becomes necessary as a source of P. The current price for importing DAP is NRs 114 per kg, while the sales price is NRs 44 per kg. To make DAP more affordable for farmers, a subsidy of NRs 70 per kg is provided [9]. Without this subsidy, fertilizer would not be able to afford the high price of DAP. A significant portion of the agricultural budget (approximately 30%) is allocated to fertilizer subsidies [10]. However, despite the allocation of funds, farmers often do not receive an adequate supply of fertilizers on time. Therefore, finding a sustainable solution to this issue is crucial.

In the soils, P exists as both organic (Po), as well as, inorganic (Pi) forms [11]. Most soils have a high capacity to fix P, and the low P use efficiency of most crops (around 10–15%) leads to the accumulation of surplus P fertilizer in various inorganic and organic forms in the soil [12, 13]. This accumulated P stock, known as legacy P, has the potential to play a crucial role in maintaining agricultural productivity with lower P input requirements, provided that crops can efficiently access this P [14]. It has even been suggested that the accumulated P in agricultural soils would be sufficient to sustain maximum crop yields worldwide for about 100 years if it were readily available [15]. The P accumulation depends on various factors such as parent material, climate, soil texture, vegetation cover, and soil management practices [16]. The amount and spatial variation of soil P are highly variable and not only influenced by soil-forming factors like parent material, climate and time, but also by land use types and management practices in the agricultural soil ecosystem [17, 18].

The determination of organic and inorganic P pools is made possible through the sequential fractionation method. P fractionation has been investigated since 1957 and is considered an applicable technique for determining the status of P in soils, as well as studying its chemistry and genesis [19, 20]. Sequential fractionation methods to determine soil P fractions have proven to be valuable in providing information on the availability and dynamics of soil P, and as a result, they have been widely used to date [21, 22].The sequential P fractionation method, enables various inorganic P fractions to be extracted separately, such as loosely bound soluble P, Al-P, Fe-P, Reductant soluble P, and Ca-P. Additionally, organic P fractions, including labile P, moderately labile P, fluvic acid P (moderately resistant), humic acid P (highly resistant), and non-labile P, can also be extracted [22].

In Nepal, there is a high variation in soil pH and a diverse proportion of soil separates, which are common soil features [23, 24]. These properties greatly affect P availability in the soils. According to Dawadi and Thapa [25], 53% of the tested soil samples at the Central Agricultural Laboratory, Hariharbhawan, Lalitpur were found to be acidic in nature. Additionally, most of the acidic soil pockets are concentrated in the mid-hills region of Nepal.

There is a lack of available information regarding the different P pools in Nepal. The determination and characterization of various forms of P in Nepalese soils have not received significant attention. In light of this, a present study was conducted to assess the effect of cropping systems on soil P fractions in the central mid-hills of Nepal.

## Materials and methods

### Study area

**Fig 1:**
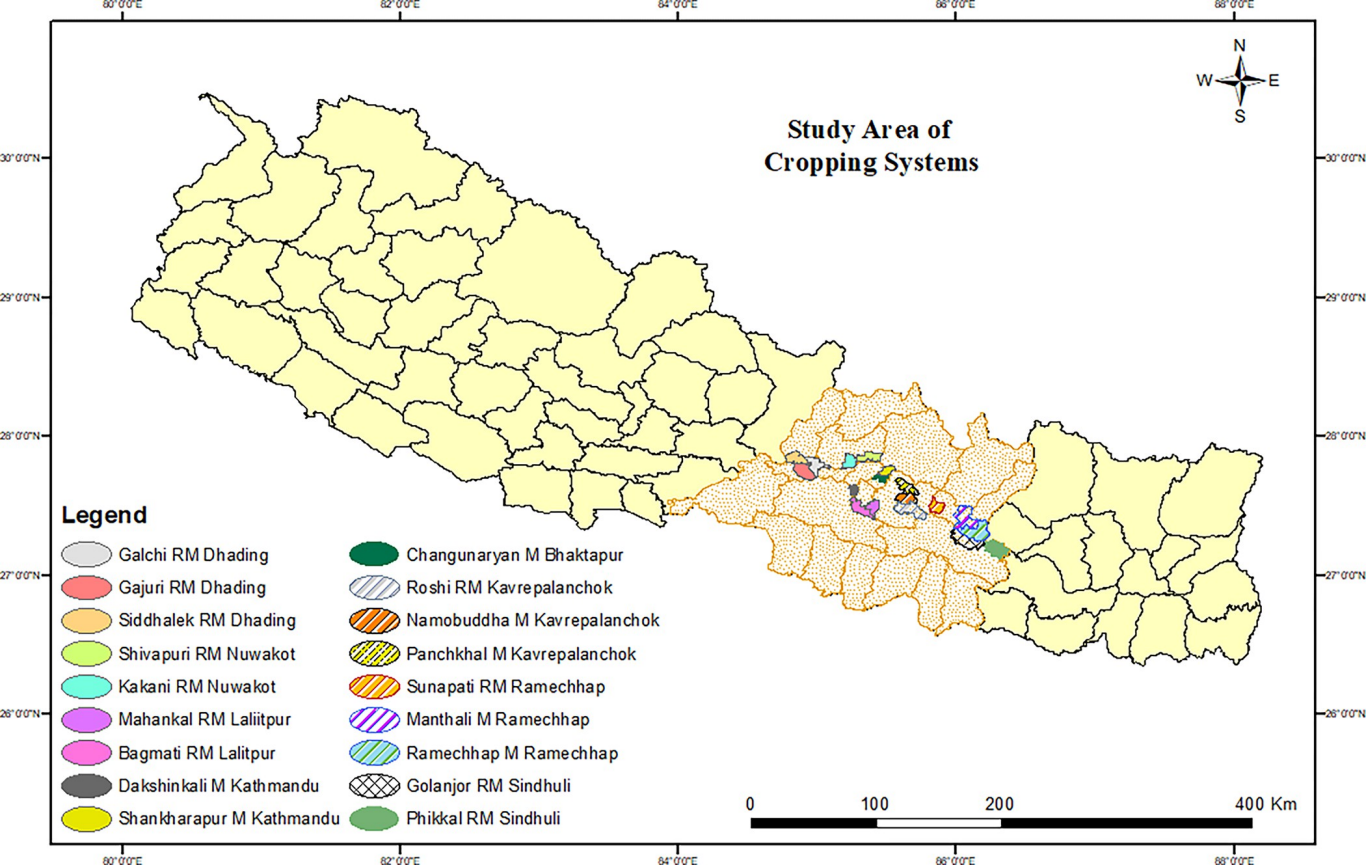
Location map of the study area (source of base map: National spatial data center under survey department, Nepal (https://nationalgeoportal.gov.np/#/)).

The study was conducted in the central mid-hill region of Nepal (Fig 1). The area is characterized by the cultivation of various crops such as maize, wheat, rice, millet, buckwheat, etc., as well as a variety of vegetables including tomato, cabbage, cauliflower, cucumber, pumpkin, chilli, radish, carrot, etc. Additionally, a range of fruits such as orange, sweet orange, kiwi, litchi, mango, avocado, dragon fruit, guava, etc., were commonly grown in the fields. The study sites were situated in the sub-tropical climatic zone, with cool winters and mild summers in terms of temperature, although the valley and river basin areas experienced relatively hotter conditions.

### Cropping systems

Among the different cropping systems adopted in the central mid-hills region of Nepal, four specific systems were selected: vegetable-based, fruit-based, rice-based, and maize-based systems. The nutrient sources utilized by the farmers in the selected cropping systems were documented through field questionnaires. The percentage distribution of nutrient sources applied in each cropping system is presented in Table 1. Various nutrient sources were employed by the farmers. In the fruit-based system, these included FYM, compost, poultry manure, goat manure, urea, DAP, MOP, and borax. Similarly, the vegetable-based system involved the application of FYM, compost, vermicompost, poultry manure, goat manure, urine, oilcake, urea, DAP, MOP, borax, zinc sulphate, and fertilizers containing calcium. Furthermore, the rice-based system incorporated FYM, compost, poultry manure, goat manure, urea, DAP, MOP, and zinc sulphate. In the maize-based system, the nutrient sources encompassed FYM, poultry manure, goat manure, urea, and DAP. Notably, the types of organic and inorganic sources, their application timings, and the quantities utilized exhibited variations across the different cropping systems.

**Table 1:** The percentage distribution of nutrient sources applied by farmers in the selected cropping systems.

Cropping Systems	Organic	Inorganic	Organic+Inorganic	Without nutrient source
Vegetable-based	33.33%	0	66.67%	0
Fruit-based	58.33%	1.67%	26.67%	13.33%
Rice-based	3.33%	6.67%	90.00%	0
Maize-based	13.33%	1.67%	85.00%	0

### Soil sampling and processing

The soil samples were collected from a depth of 0-20cm using a soil sampling auger. The geographical positioning of each soil sampling point was determined using a handheld GPS device. A total of 240 samples (60 samples for each cropping system factor level) were collected from various sites in the central mid-hill region, as depicted in Fig 1.

For the determination of general physicochemical parameters and inorganic P fractions, the air-dried soil samples were ground and sieved through a 2 mm sieve to obtain a homogenous sample. On the other hand, for the determination of organic fractionations, fresh soil samples were kept at 4°C in a refrigerator until the start of analysis.

### Laboratory analysis

The soil texture was determined using the hydrometer method [26]. The pH was measured in distilled water (1:2) using the potentiometric method [27]. Electrical conductivity was determined in distilled water (1:5) [28]. Cation exchange capacity (CEC) was measured using the sodium acetate method [29]. Calcium carbonate (CaCO_3_) content was determined using the titrimetric method [30]. Organic matter content was determined using the Walkley and Black [31] method. Olsen P was measured using the modified Olsen [32] method. Bray-1 P was determined using the Bray and Kurtz [33] method. Mehlich-3 P was measured using the Mehlich [34] method.

Total P was determined using the sodium carbonate fusion method followed by spectrophotometric measurement, as outlined by Smith and Bain [35]. In addition to total P, the various organic and inorganic P fractions were sequentially fractioned using the chemical fractionation method described by Zhang and Kovar [22]. The residual P was then calculated by subtracting the sum of the organic and inorganic fractions from the total P, as suggested by Fanjana et al. [36], Nishigaki et al. [37], and Wierzbowska et al. [38].

### Statistical analysis

A one-way analysis of variance (ANOVA) was conducted using R programming to examine the impact of cropping systems on P fractions and related attributes. The homogeneity of variance between factor levels was assessed using Levene’s test [39]. If the variance was found to be equal, Fisher’s ANOVA [40] was employed. However, if the variance was unequal, Welch’s ANOVA [41] was utilized. In cases where Fisher’s ANOVA yielded significant results, a Tukey post-hoc test was conducted for multiple pairwise comparisons. For significant Welch’s ANOVA results, a Games-Howell post hoc test was performed.

## Results

### General soil parameters

#### Soil pH

**Fig 2:**
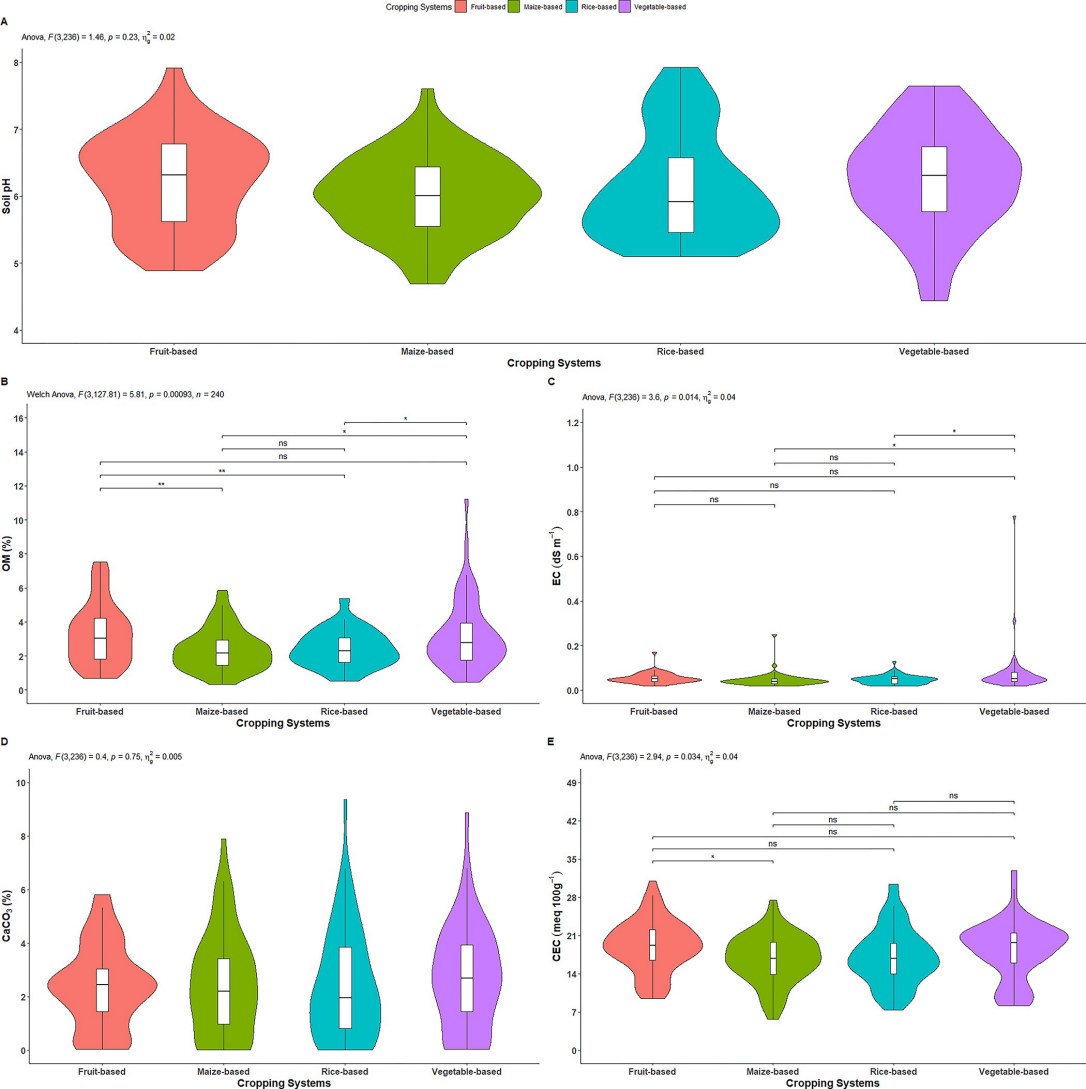
Effect of cropping systems on A) soil pH, B) organic matter, C) electrical conductivity, D) calcium carbonate, and E) cation exchange capacity. *indicates p = ≤0.05; ** indicates p = ≤0.01; *** indicates p = ≤0.001; ns = non-significant; Parameter having no any symbols indicates not statistically significant.

The cropping systems had no significant effect on soil pH (Fig 2A). As illustrated in Table 2, among the cropping systems, the vegetable-based system exhibited highest pH (6.26), followed by fruit-based (6.23) and rice-based (6.14) systems, while the lowest pH value (6.01) was recorded in maize-based cropping system.

**Table 2:** The soil physicochemical parameters as influenced by cropping systems.

Cropping Systems	pH	OM	EC	CaCO_3_	CEC	Sand	Silt	Clay
		%	dS m^-1^	%	meq 100g^-1^	%		
Vegetable-based	6.26	3.21	0.075	2.72	18.38	36.25	41.34	22.41
Fruit-based	6.23	3.29	0.054	2.38	19.06	36.77	41.51	21.73
Rice-based	6.14	2.43	0.047	2.41	17.09	35.22	43.86	20.92
Maize-based	6.01	2.38	0.046	2.46	16.72	39.09	39.75	21.16

Min., Minimum; Max., Maximum; OM, Organic matter; EC, Electrical conductivity; CaCO_3_, Calcium carbonate; CEC, Cation exchange capacity

#### Organic matter

The soil organic matter (OM) was significantly affected by cropping systems (Fig 2B). As depicted in Table 2, the fruit-based system exhibited highest OM (3.29%), followed by vegetable-based (3.21%), and rice-based (2.43%) systems. On the other hand, maize- based system had the lowest OM (2.38%). The OM observed in vegetable-based system was statistically similar to that of the fruit-based system. Likewise, the maize-based system and the rice-based system exhibited statistically similar OM level. However, the fruit-based system had a significantly higher OM content compared to both the maize-based and rice-based systems. Additionally, the vegetable-based system also demonstrated a statistically higher OM content compared to both the rice-based and maize-based systems.

#### Electrical conductivity

The cropping systems had a significant effect on the electrical conductivity (EC) of the soils (Fig 2C). As depicted in Table 2, the highest EC (0.075 dS m^-1^) was determined in vegetable-based system, followed by fruit-based (0.054 dS m^-1^) and rice-based (0.047 dS m^-1^) systems. The lowest EC (0.046 dS m^-1^) was recorded in the maize-based system. The EC observed in the fruit-based system was statistically on par with the vegetable-based, rice-based and maize-based systems. Moreover, the maize-based and rice-based systems were also statistically similar. On the other hand, vegetable-based system had a statistically higher EC than rice-based and maize-based systems.

#### Calcium carbonate

The effect of the cropping systems on calcium carbonate (CaCO_3_) was not statistically significant (Fig 2D). As illustrated in Table 2, the highest CaCO_3_ (2.72%) was observed in vegetable-based system, followed by maize-based (2.46%) and rice-based (2.41%) systems, while the lowest CaCO_3_ (2.38%) was recorded in fruit-based system.

### Cation exchange capacity

The cropping systems had a significant effect on cation exchange capacity (CEC) was significantly affected by cropping systems (Fig 2E). As shown in Table 2, the fruit-based system exhibited the highest CEC (19.06 meq 100g^-1^), followed by vegetable-based (18.38 meq 100g^-1^) and rice-based (17.09 meq 100g^-1^) systems. The maize-based system had the lowest CEC (16.72 meq 100g^-1^). The CEC in the fruit-based system was significantly higher than in maize-based system, while all other cropping systems had statistically similar values.

### Soil separates

**Fig 3:**
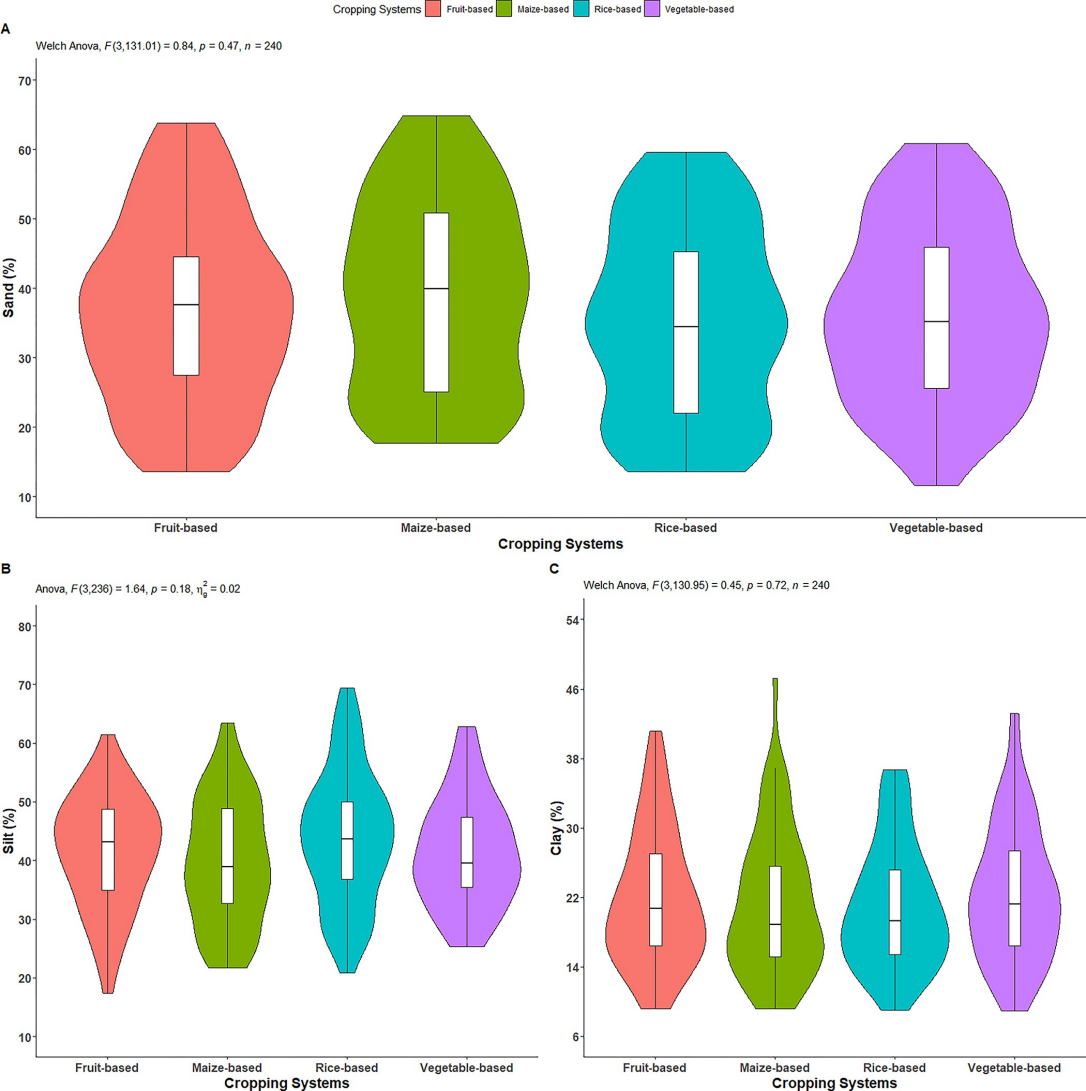
Effect of cropping systems on A) sand separate, B) silt separate, and C) clay separate. *indicates p = ≤0.05; ** indicates p = ≤0.01; *** indicates p = ≤0.*001; ns = non-significant;* Parameter having no any symbols indicates not statistically significant.

The effect of the cropping systems on the proportion of sand separate was not statistically significant (Fig 3A). As shown in Table 2, the highest content (39.09%) was observed in maize-based system, followed by fruit-based (36.77%) and vegetable-based (36.25%) systems, while the lowest content (35.22%) was in rice-based system.

Similar to sand separate, the effect of cropping systems on proportion of silt separate was not statistically significant (Fig 3B). As depicted in Table 2, the highest proportion (43.86%) was observed in rice-based system, followed by fruit-based (41.51%) and vegetable-based (41.34%) systems, while the lowest proportion (39.75%) was found in maize-based system.

**Fig 4:**
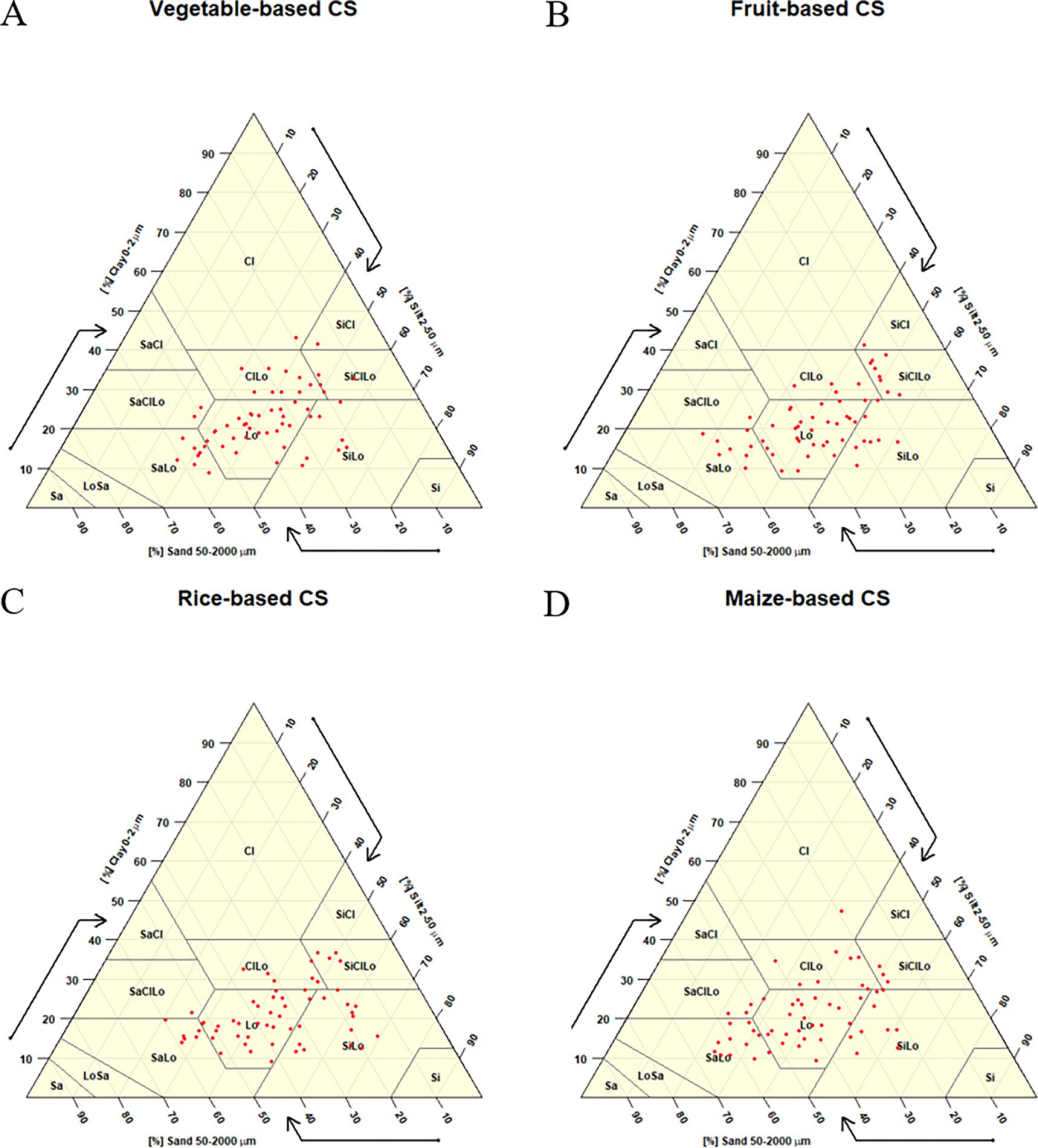
Distribution of soil samples in textural class on A) vegetable-based, B) fruit-based, C) rice-based and D) maize-based cropping systems.

Similar to other separates, the effect of cropping systems on proportion of clay separate was not statistically significant (Fig 3C). As illustrated in Table 2, the highest proportion (22.14%) was observed in vegetable-based system, followed by fruit-based (21.73%) and maize-based (21.16%) systems, while the lowest proportion (20.92%) was found in rice-based system. The different textural class observed in the cropping systems is shown in the Fig 4.

#### Olsen P

**Fig 5:**
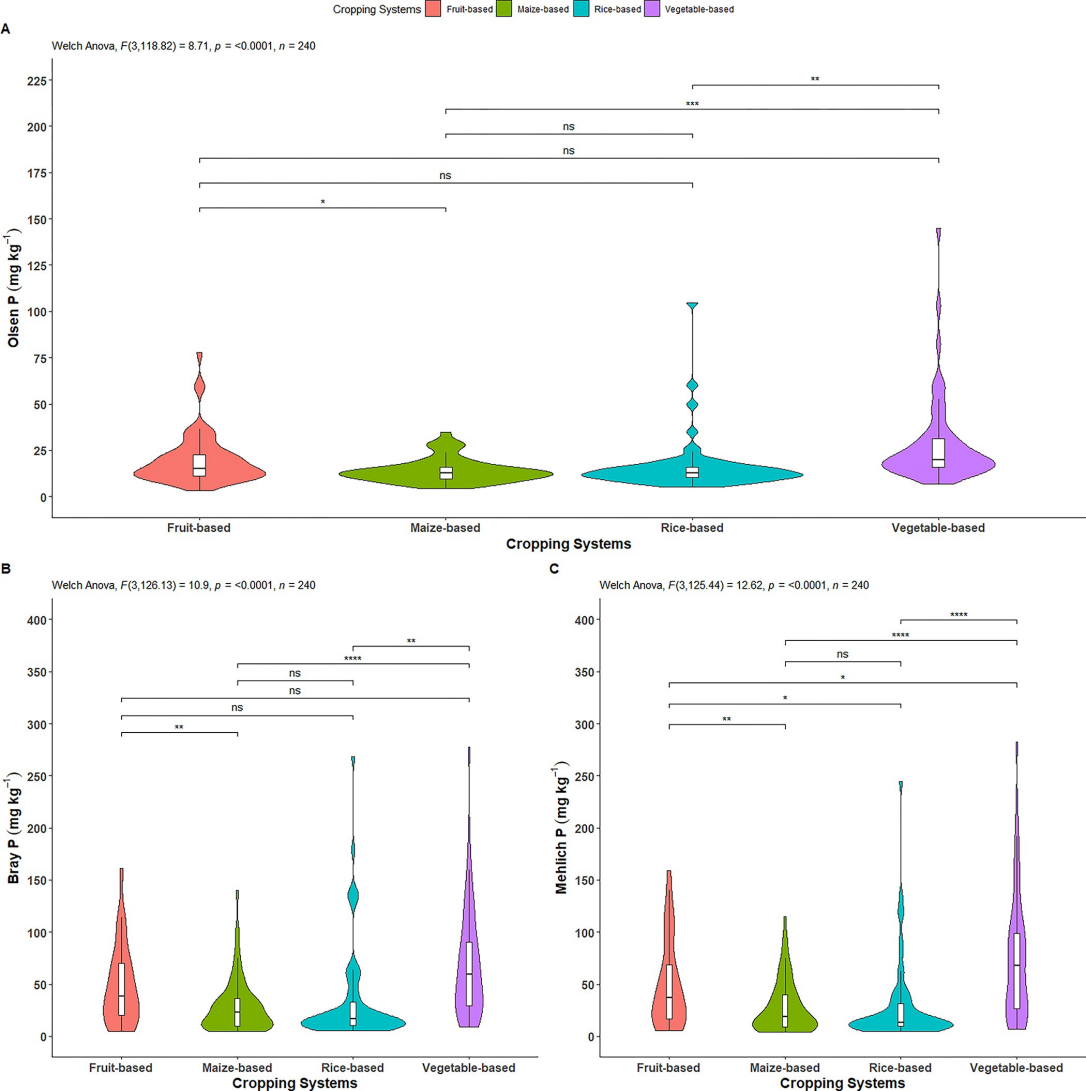
Effect of cropping systems on A) Olsen P, B) Bray-1 P and C) Mehlich-3 P. *indicates p = ≤0.05; ** indicates p = ≤0.01; *** indicates p = ≤0.001; ns = non-significant. Parameter having no any symbols indicates not statistically significant.

The Olsen P was significantly influenced by cropping systems (Fig 5A). As shown in Table 3 and Fig 5A, the vegetable-based system had a significantly higher Olsen P (28.39 mg kg^-1^) compared to rice-based (16.21 mg kg^-1^) and maize- based (14.10 mg kg^-1^) systems. Furthermore, the fruit-based system showed a significantly higher Olsen P (19.80 mg kg^-1^) compared to maize-based system. However, fruit-based system had similar effect on Olsen P compared to rice- based and vegetable-based systems. Similarly, rice-based and maize-based systems had a non-significant effect on Olsen P. The highest proportion of total P for Olsen P (1.23%) was observed in the vegetable-based system, while the lowest (0.65%) was in the maize-based system (Table 3).

**Table 3:** The proportion of total P for Olsen, Bray-1 and Mehlich-3 P as influenced by cropping systems.

Cropping Systems	Olsen P	Bray-1 P	Mehlich-3 P	Proportion of total P		
				Olsen P	Bray-1 P	Mehlich-3 P
	mg kg^-1^			%		
Vegetable-based	28.39	69.16	74.34	1.23	3.00	3.23
Fruit-based	19.80	47.81	49.59	0.88	2.14	2.15
Rice-based	16.21	34.84	29.07	0.78	1.67	1.26
Maize-based	14.10	28.69	28.28	0.65	1.33	1.23

Min., Minimum; Max., Maximum

#### Bray-1 P

Similar to Olsen P, Bray-1 P was also significantly affected by cropping systems (Fig 5B). As depicted in Table 3 and Fig 5B, the vegetable-based system exhibited was a significantly higher Bray-1 P (69.16 mg kg^-1^) compared to maize-based (28.69 mg kg^-1^) and rice-based (34.84 mg kg^-1^) systems. Additionally, the fruit-based system also had a significantly higher Bray-1 P (47.81 mg kg^-1^) compared to the maize-based system. However, fruit-based system showed similar effects on Bray-1 P compared to the rice-based and vegetable-based systems. Similarly, the rice-based and maize-based systems had also similar effects on Bray-1 P. The highest proportion of total P for Bray-1 P (3.00%) was observed in the vegetable-based system, while the lowest (1.33%) was in the maize-based system (Table 3).

#### Mehlich-3 P

Similar to the previous available P measurements, Mehlich-3 P was also significantly affected by the cropping systems (Fig 5C). As shown in Table 3 and Fig 5C, the highest Mehlich-3 P (74.34 mg kg^-1^) was observed in vegetable-based system, followed by fruit-based (49.59 mg kg^-1^) and rice-based (29.07 mg kg^-1^) systems. The lowest content (28.28 mg kg^-1^) was in maize-based system. There were significant differences in Mehlich-3 P content among all systems, except between the rice-based and maize-based systems. The highest proportion of total P for Mehlich-3 P (3.23%) was observed in the vegetable-based system, while the lowest (1.23%) was in the maize-based system (Table 3).

### Soil inorganic P fractions

#### Loosely bound P

**Fig 6:**
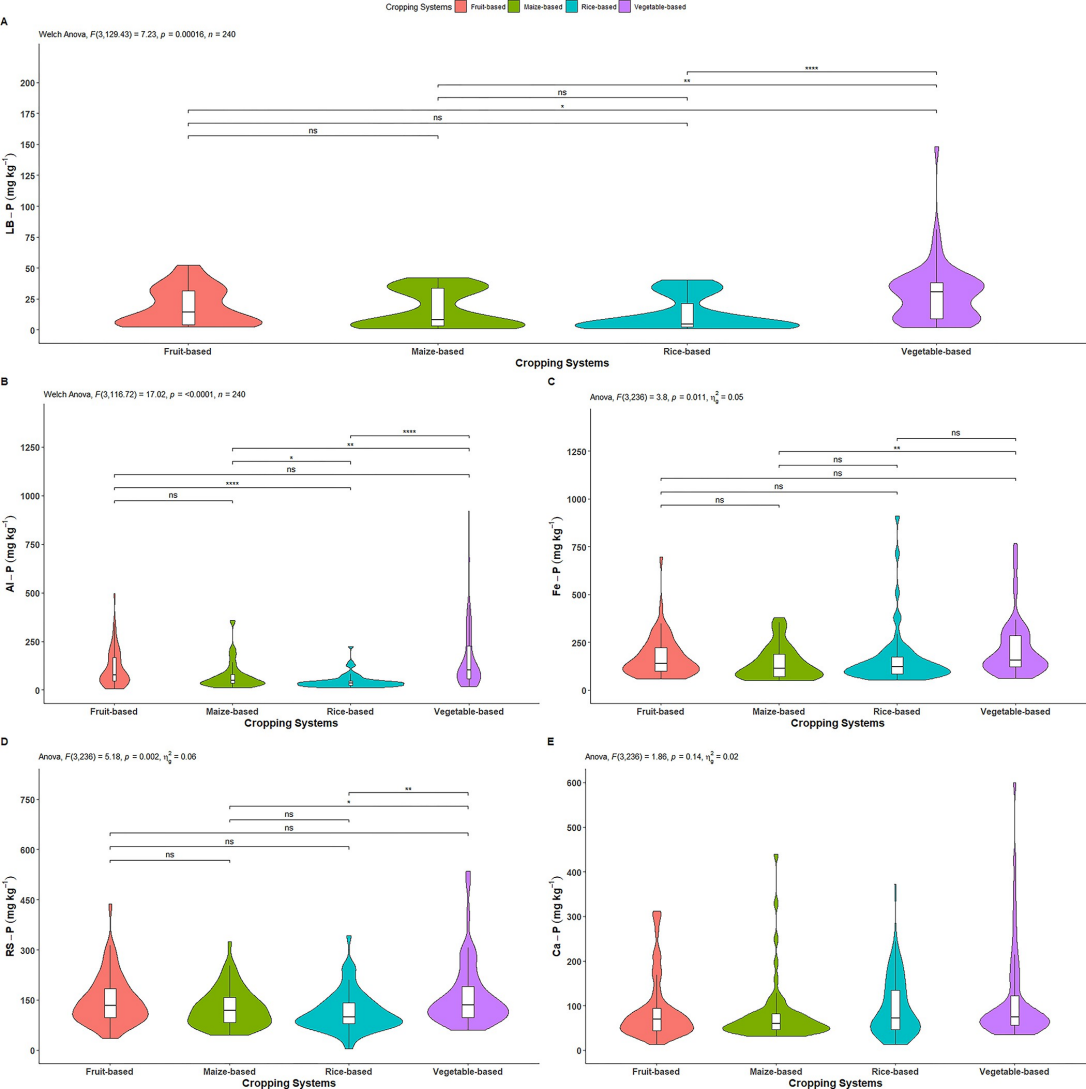
Effect of cropping systems on A) loosely bound P, B) aluminum P, C) iron P, D) reductant soluble P and E) calcium P *indicates p = ≤0.05; ** indicates p = ≤0.01; *** indicates p = ≤0.001; ns = non-significant; Parameter having no any symbols indicates not statistically significant.

The loosely bound P was significantly affected by cropping systems (Fig 6A). As shown in Table 4 and Fig 6A, the vegetable-based system had a significantly higher loosely bound P (28.41 mg kg^-1^) compared to the fruit-based (18.41 mg kg^-1^), maize-based (16.20 mg kg^-1^), and rice-based (12.17 mg kg^-1^) systems. In contrast, the fruit-based, maize-based and rice-based systems had showed similar effects on loosely bound P. The highest fractional distribution of loosely bound P (1.23%) was observed in the vegetable-based system, while the lowest (0.58%) was found in the rice-based system (Table 4).

**Table 4:** The fractional distribution of inorganic P fractions as influenced by cropping systems.

Cropping Systems	Inorganic P fractions					Fractional distribution				
	LB-P	Al-P	Fe-P	RS-P	Ca-P	LB-P	Al-P	Fe-P	RS-P	Ca-P
	mg kg^-1^					%				
Vegetable-based	28.41	167.65	218.03	164.08	113.77	1.23	7.27	9.46	7.12	4.94
Fruit-based	18.47	111.67	172.05	149.29	91.22	0.83	4.99	7.69	6.67	4.08
Rice-based	12.17	45.08	163.19	115.75	97.43	0.58	2.17	7.84	5.56	4.68
Maize-based	16.20	73.94	141.33	126.83	79.89	0.75	3.42	6.54	5.87	3.70

Min., Minimum; Max., Maximum; LB-P, Loosely bound P; Al-P, Aluminum bound P; Fe-P, Iron bound P; RS-P, Reductant soluble P; Ca-P, Calcium bound P

#### Aluminum P

The aluminum P (Al-P) was significantly affected by cropping systems (Fig 6B). As depicted in Table 4 and Fig 6B, there was a significant difference in Al-P content among the vegetable-based (167.65 mg kg^-1^), maize-based (73.94 mg kg^-1^) and rice-based (45.08 mg kg^-1^) systems. The fruit-based system had a significantly higher Al-P (111.67 mg kg^-1^) compared to rice-based system, but it was statistically similar to vegetable-based and maize-based systems. The highest fractional distribution of Al-P (7.27%) was observed in the vegetable-based system, while the lowest (2.17%) was found in the rice-based system (Table 4).

#### Iron P

The iron P (Fe-P) was significantly affected by cropping systems (Fig 6C). As illustrated in Table 4 and Fig 6C, Fe-P observed in the vegetable-based system was significantly higher (218.03 mg kg^-1^) than in maize-based system (141.33 mg kg^-1^), while it was statistically similar to the fruit-based (172.05 mg kg^-1^) and rice-based (163.19 mg kg^-1^) system. However, Fe-P content in the fruit-based, rice-based and maize-based systems were statistically similar to each other. The highest fractional distribution of Fe-P (9.46%) was observed in the vegetable-based system, while the lowest (6.54%) was found in the maize-based system (Table 4).

#### Reductant soluble P

The reductant soluble P was significantly affected by cropping systems (Fig 6D). As illustrated in Table 4 and Fig 6D, reductant soluble P was significantly higher in the vegetable-based system (164.08 mg kg^-1^) compared to maize-based system (126.83 mg kg^-1^) and rice-based system (115.75 mg kg^-1^), while it was similar to the fruit-based system (149.29 mg kg^-1^). However, the fruit-based system, maize-based system and rice-based system showed a non-significant effect among each other. The highest fractional distribution of reductant soluble P (7.12%) was observed in the vegetable-based system, while the lowest (5.56%) was found in the rice-based system (Table 4).

#### Calcium P

The effect of cropping systems on calcium P (Ca-P) was non-significant (Fig 6E). As shown in Table 4 and Fig 6E, the highest calcium P was observed in vegetable-based system (113.77 mg kg^-1^), followed by rice-based (97.43 mg kg^-1^), fruit-based (91.22 mg kg^-1^) and maize-based (79.89 mg kg^-1^) systems. The highest fractional distribution of Ca-P (4.94%) was observed in the vegetable-based system, while the lowest (3.70%) was found in the maize-based system (Table 4).

### Soil organic P fractions

#### Labile organic P

**Fig 7:**
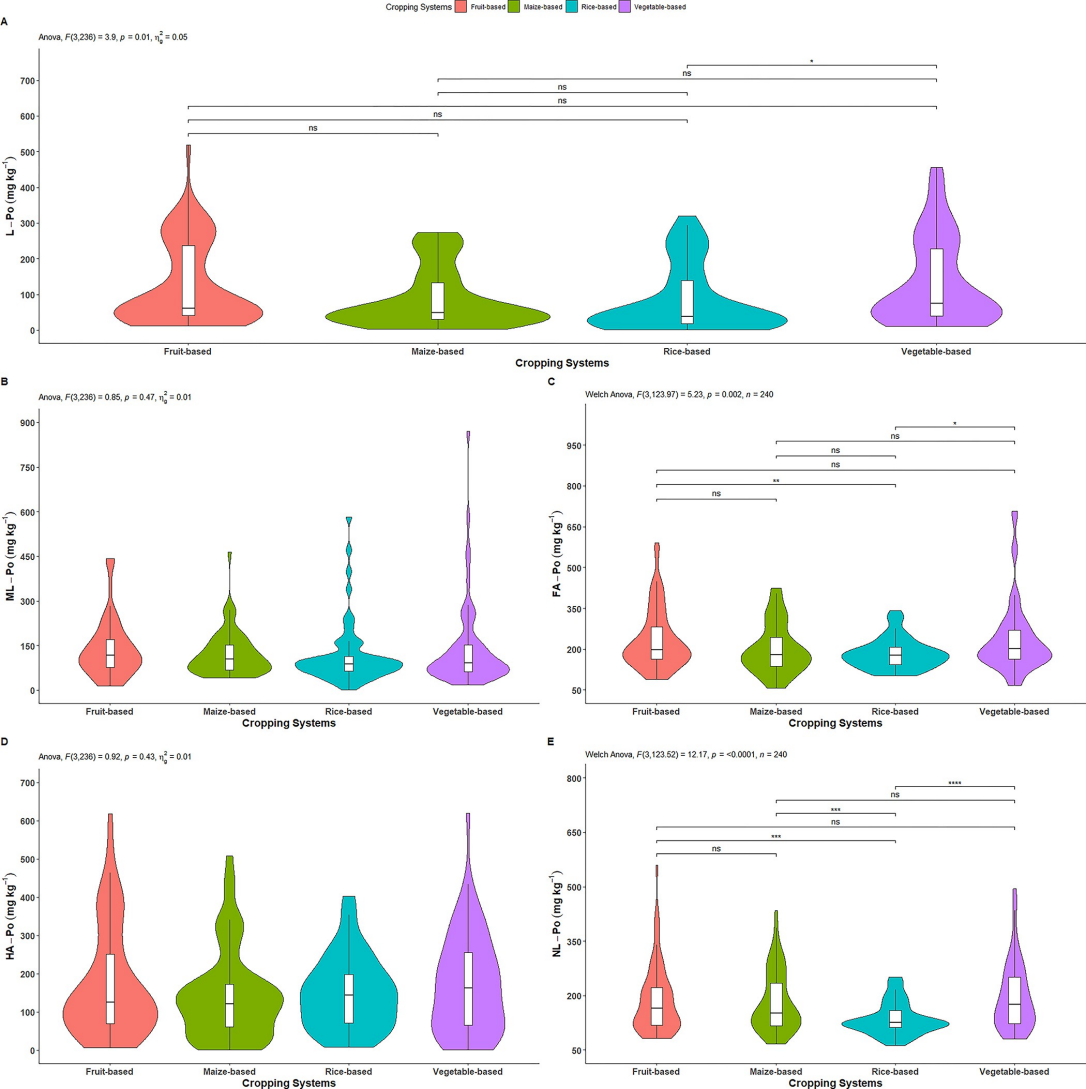
Effect of cropping systems on A) labile organic P, B) moderately labile organic P, C) fluvic acid organic P, D) humic acid organic P and E) non-labile organic P. *indicates p = ≤0.05; ** indicates p = ≤0.01; *** indicates p = ≤0.001; ns = non-significant; Parameter having no any symbols indicates not statistically significant.

The labile organic P (L-Po) was significantly affected by cropping systems (Fig 7A). As illustrated in Table 5 and Fig 7A, the highest L-Po was observed in vegetable-based system (133.30 mg kg^-1^), followed by fruit-based (125.45 mg kg^-1^), maize-based (86.65 mg kg^-1^) and rice-based (81.09 mg kg^-1^) systems. The labile Po in vegetable-based system was significantly higher than in the rice-based system, while it was statistically similar in other cropping systems. Moreover, fruit-based, maize-based and rice-based systems were non-significantly differences among each other. The highest fractional distribution of L-Po (5.78%) was observed in the vegetable-based system, while the lowest (3.90%) was found in the rice-based system (Table 5).

**Table 5:** The fractional distribution of organic P fractions as influenced by cropping systems.

Cropping Systems	Organic P fractions					Fractional distribution				
	L-Po	ML-Po	FA-Po	HA-Po	NL-Po	L-Po	ML-Po	FA-Po	HA-Po	NL-Po
	mg kg^-1^					%				
Vegetable-based	133.30	142.31	235.75	171.88	198.24	5.78	6.17	10.23	7.46	8.60
Fruit-based	125.45	139.66	234.67	173.10	188.92	5.61	6.24	10.49	7.74	8.44
Rice-based	81.09	116.02	183.05	149.16	135.87	3.90	5.58	8.80	7.17	6.53
Maize-based	86.65	121.57	201.28	143.64	183.03	4.01	5.63	9.32	6.65	8.47

Min., Minimum; Max., Maximum; L-Po, Labile organic P; ML-Po, Moderately labile organic P; FA-Po, Fluvic acid organic P; HA-Po, Humic acid organic P; NL-Po, Non-labile organic P

#### Moderately labile organic P

The moderately labile organic P (ML-Po) was not significantly affected by cropping systems (Fig 7B). As shown in Table 5 and Fig 7B, the highest content of ML-Po was observed in vegetable-based system (142.31 mg kg^-1^), followed by fruit-based (139.66 mg kg^-1^), maize-based (121.57 mg kg^-1^) and rice-based (116.02 mg kg^-1^) systems. The highest fractional distribution of ML-Po (6.24%) was observed in the fruit-based system, while the lowest (5.58%) was found in the rice-based system (Table 5).

#### Fluvic acid organic P

The fluvic acid organic P (FA-Po) was significantly affected by cropping systems (Fig 7C). As shown in Table 5 and Fig 7C, the highest content of FA-Po was determined in vegetable-based system (235.75 mg kg^-1^), followed by fruit-based (234.67 mg kg^-1^), maize-based (201.28 mg kg^-1^) systems, and the lowest in rice-based system (183.05 mg kg^-1^). The FA-Po in rice-based system was significantly lower than in the vegetable-based and fruit-based systems, while it was statistically similar to the maize-based system. Similarly, the FA-Po content was statistically similar among the vegetable-based, fruit-based and maize-based systems. The highest fractional distribution of FA-Po (10.49%) was observed in the fruit-based system, while the lowest (8.80%) was found in the rice-based system (Table 5).

#### Humic acid organic P

The humic acid organic P (HA-Po) was not significantly influenced by cropping systems (Fig 7D). As illustrated in Table 5 and Fig 7D, the highest HA-Po was observed in fruit-based system (173.10 mg kg^-1^), followed by vegetable-based (171.88 mg kg^-1^), rice-based (149.16 mg kg^-1^) and maize-based (143.64 mg kg^-1^) systems. The highest fractional distribution of HA-Po (7.74%) was observed in the fruit-based system, while the lowest (6.65%) was found in the maize-based system (Table 5).

#### Non-labile organic P

The non-labile organic P (NL-Po) was significantly affected by cropping systems (Fig 7E). As shown in Table 5 and Fig 7E, the NL-Po observed in rice-based system (135.87 mg kg^-1^) was significantly lower than in the vegetable-based (198.24 mg kg^-1^), fruit-based (188.92 mg kg^-1^) and maize-based (183.03 mg kg^-1^) systems. However, there was no significant difference in NL-Po content among the vegetable-based, fruit-based, and maize-based systems. The highest fractional distribution of NL-Po (8.60%) was observed in the vegetable-based system, while the lowest (6.53%) was found in the rice-based system (Table 5).

#### Residual P fraction

**Fig 8:**
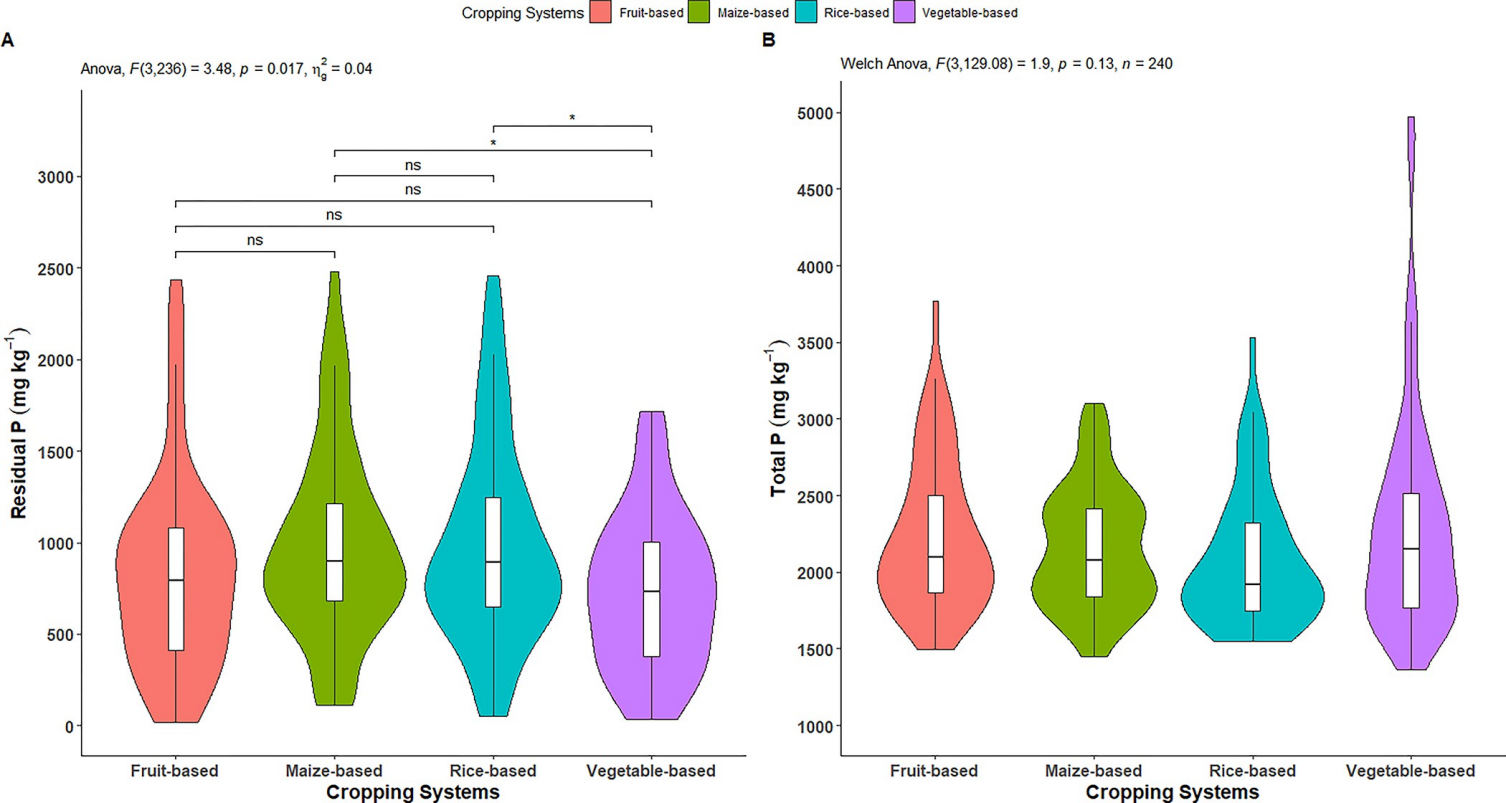
Effect of cropping systems on A) residual P, and B) total P. *indicates p = ≤0.05; ** indicates p = ≤0.01; *** indicates p = ≤0.001; ns = non-significant; Parameter having no any symbols indicates not statistically significant.

The residual P was significantly affected by cropping systems (Fig 8A). As shown in Table 6 and Fig 8A, the highest residual P was observed in maize-based system (986.15 mg kg^-1^), followed by rice-based (981.85 mg kg^-1^) and fruit-based (833.53 mg kg^-1^) systems, while lowest was in vegetable-based system (731.53 mg kg^-1^). The residual P content in vegetable-based system was significantly lower than in maize-based and rice-based systems, but statistically similar to the fruit-based system. Furthermore, the residual P content in maize-based, rice-based, and fruit-based systems were statistically similar. The highest fractional distribution of residual P (47.19%) was observed in the rice-based system, while the lowest (31.74%) was found in the vegetable-based system (Table 6).

**Table 6:** The fractional distribution of residual P fraction as influenced by cropping systems.

Cropping Systems	Residual P	Fractional distribution	Total P
	mg kg^-1^	%	mg kg^-1^
Vegetable-based	731.53	31.74	2304.94
Fruit-based	833.18	37.23	2237.65
Rice-based	981.85	47.19	2080.67
Maize-based	986.15	45.64	2160.52

Min., Minimum; Max., Maximum

#### Total P

The effect of cropping systems on total P was non-significant (Fig 8B). As mentioned in Table 6 and Fig 8B, the highest total P was observed in vegetable-based system (2304.94 mg kg^-1^), followed by fruit-based (2237.65 mg kg^-1^) and maize-based (2160.52 mg kg^-1^) systems, while lowest was recorded in the rice-based system (2080.67 mg kg^-1^).

## Discussion

### General soil parameters

Soil pH is a key chemical parameter that governs soil nutrient availability, soil microbial activities, and crop growth and development [42]. A wide range of soil pH values was observed, ranging from highly acidic to moderately alkaline; however, the majority of samples fell within the acidic range. The variation in soil pH can be attributed to several factors, including differences in parent material [43], topography, slope [44], and variations in soil management practices.

Soil OM represents the entirety of organic material present in the soils [45]. The fruit-based system exhibits a high OM content, which can be attributed to several factors, including the regular accumulation of fallen leaves, the presence of surface cover due to the plant canopy, minimal soil disturbance, and the annual addition of organic manure. Similarly, the vegetable-based system demonstrates a comparatively high OM content, likely due to the regular application of organic manure prior to planting the majority of vegetables.

In contrast, the maize-based upland system relies on organic manure application once a year before maize sowing, although the amount applied is relatively less than in the fruit-based and vegetable-based systems. Similarly, in the rice-based lowland system, farmers typically apply organic manure once a year, usually during winter or summer. However, some fields located far away from organic nutrient sources do not receive regular or sufficient amounts of organic manure. Consequently, the maize-based and rice-based systems exhibit lower OM content compared to the fruit-based and vegetable-based systems. Previous studies have reported that the addition of organic manure, along with the accumulation of fallen leaves and the presence of canopy cover, can enhance OM content in soils [46–48].

Soil EC is commonly employed as an indicator of soil salinity, particularly in specific soil types [49]. The recorded EC values ranged from 0.02 to 0.78 dS m^-1^, falling within the normal range for plant growth, as suggested by Bajwa et al. [50]. Significantly higher EC levels were observed in the vegetable-based system compared to the rice-based and maize-based systems. This disparity might be attributed to the greater application of salinity-causing ions through organic and inorganic nutrient sources specific to each cropping system. Previous studies conducted by Ropi et al. [51] and Gikonyo et al. [52] have reported that soil management practices involving the accumulation of ions such as chloride, sulfate, potassium, sodium, and nitrate can result in an increase in soil EC.

The presence of CaCO_3_ in soils serves as a cementing agent, enhancing soil aggregation and structure [53]. The relatively lower levels of CaCO_3_ observed in soils may be attributed to the predominance of non-calcareous parent material. This finding is supported by the soil pH data, where a higher proportion of samples exhibiting acidic characteristics rather than alkaline.

Cation exchange capacity (CEC) measures a soil’s ability to retain and exchange cations, which is crucial for nutrient availability and plant growth [54]. Overall, the CEC values ranged from 5.67 to 32.88 meq 100g^-1^. Based on the CEC classification provided by Yunan et al. [54], the observed CEC values indicate a range of fertility levels, ranging from low (<10 meq 100g^-1^) to high (>20 meq 100g^-1^). The comparatively higher CEC observed in the fruit-based system could be attributed to the greater organic matter content present in that cropping system. Organic matter serves as a source of negative charge for cation adsorption, leading to an increase in CEC in the soils [55–57].

Soil texture refers to the relative proportions of sand, silt, and clay particles present in the soil [58]. The variation in soil particle size is primarily attributed to the parent material and the degree of weathering [59]. Therefore, no significant differences were observed in soil texture between the cropping systems. Overall, the soils exhibit a textural class ranging from moderately coarse to fine, with the medium textural class being dominant across all cropping systems, as reported by Weil and Brady [59].

Olsen P, Bray I P, and Mehlich III P are indicators of the available P content in soils [60]. The higher application of organic and inorganic nutrient sources at the beginning of each crop in the vegetable-based system might be responsible for the high levels of available P in the soils. Previous studies have demonstrated that increased application of organic and inorganic nutrient sources leads to enhanced P accumulation in the soils [51, 61]. The application of organic manure releases organic acids that act as chelating agents for P-fixing cations, such as aluminum, iron, calcium, and magnesium, thereby increasing P availability [62, 63].

### Inorganic P fractions

The fractional distribution of total P showed low levels of loosely bound P, which represents the inorganic P pool dissolved in water/soil solution and readily available for plant uptake [64]. This may be attributed to rapid uptake by plants or microorganisms, or the formation of secondary mineral phases [65]. However, the vegetable-based system exhibited significantly higher levels of loosely bound P. This could be attributed to the more intensive cultivation with comparatively more nutrient sources compared to other cropping systems. It is well-known that increasing concentrations of applied P, whether from organic or inorganic sources, generally enhance their mobility and content in soils [66, 67].

Aluminum-P (Al-P) refers to the inorganic P pools that form when inorganic P binds with aluminum, either as dissolved ions, oxides, or hydrous oxide [59]. This pool of P is considered to be slowly available [68]. In the vegetable-based system, a higher content of Al-P was observed, which could be attributed to the intensive application of organic and inorganic nutrient sources supplying more phosphate ions, creating a favorable environment for the formation of the Al-P fraction. The presence of reactive aluminum in the soil allows it to bind to additional phosphate ions, forming a covalent Al-P bond on the surface of aluminum oxide after fertilizer application from different sources [69, 70]. On the other hand, in the rice-based system, the lower Al-P content might be attributed due to the relatively lower application of P fertilizer through organic and inorganic nutrient sources, as P uptake is higher in rice and wheat.

Iron-P (Fe-P) refers to the inorganic P fraction that forms when inorganic P binds with iron, either as dissolved ions, oxides, or hydrous oxide [59]. This fraction is considered to represent slowly available P [68]. In the vegetable-based system, a higher content of Fe-P was observed, which could be attributed to the intense application of organic and inorganic nutrient sources that supply more phosphate ions, creating a more favorable environment for the formation of the Fe-P fraction. The cumulative addition of phosphate ions occurs directly from chemical fertilizers and through the mineralization of organic sources, leading to increased phosphate ions in the soils [71, 72]. The Fe ions have the ability to quickly adsorb dissolved phosphate ions and transform them into intermediate forms [73].

The reductant soluble-P fraction represents an inactive form or a steady state of P [74]. This fraction is predominantly unavailable to plants [75]. In the vegetable-based system, a relatively higher content of reductant soluble-P was observed compared to the rice-based lowland and maize-based upland systems. This difference could be attributed to the higher application of P fertilizers at the beginning of each crop cycle through organic and inorganic nutrient sources. Conversely, the lower amount of phosphate ions formed due to comparatively less fertilizer application in the maize-based and rice-based systems could explain the lower levels of reductant soluble-P. Previous studies have shown that the incorporation of organic manure in combination with inorganic fertilizers increases the content of reductant soluble-P [72, 74, 76].

Calcium-P (Ca-P) is present in discrete particles [77]. When soluble P reacts with Ca ions and CaCO_3_, it leads to the formation of low-soluble Ca-P [78]. Ca-bound P is generally considered unavailable for plant uptake [79]. However, non-available P can become accessible to plants through desorption from Ca-bound compounds [80]. The low content of Ca-related ions, resulting from the acidic nature of the soils [81], might explain the absence of significant differences in Ca-P among the different cropping systems.

### Organic P fractions

Labile organic P (L-Po) represents the most unstable form of organic P fractions, characterized by its active and readily mineralizable nature [82]. L-Po fractions are available to plants within a relatively short period, ranging from days to a few weeks [83]. In our study, the vegetable-based system exhibited significantly higher levels of L-Po compared to the rice-based system. This difference might be attributed to the higher application of organic and inorganic nutrient sources in the vegetable-based system. Ahmed et al. [84] reported that the combined application of organic and inorganic nutrient sources remarkably increased labile Po. Conversely, the lower L-Po content in the rice-based system could be due to the continuous flooding conditions, which may have reduced the effectiveness of organic amendments. This finding aligns with the findings of Gaind & Singh [85].

Moderately labile organic P (ML-Po) refers to the easily mineralizable forms of organic P [86]. While ML-Po may not be immediately available to plants, it has the potential to become available in the medium term, ranging from months to a few years, through biological and physicochemical transformations [83]. In our study, the levels of ML-Po did not significantly differ among the different cropping systems.

Fluvic acid organic P (FA-Po), also known as moderately resistant organic P [87], refers to organic P that is not readily mineralized by microbes or easily absorbed by plants [86]. In our study, we observed that the rice-based lowland system had a significantly lower level of FA-Po compared to the vegetable-based and fruit-based systems. This difference might be attributed to the lower addition of organic nutrient sources and the prolonged water stagnation in the lowland area. In the vegetable-based system, higher amounts of P nutrients were applied through both organic and inorganic sources at the beginning of each vegetable crop. However, the application of inorganic P has been found to inhibit phosphorylase activity [88], thereby suppressing mineralization processes and promoting the accumulation of FA-Po

Humic acids are natural high molecular weight organic compounds that result from the decomposition of biomass residue [89]. Humic acid organic P (HA-Po), also known as highly resistant organic P [87], refers to organic P compounds with higher molecular weights that are associated with stable organic constituents. These compounds are resistant to degradation by microbial or enzymatic activity, making them a long-term source of P [86]. In our study, we found that the levels of HA-Po did not differ significantly among the different cropping systems.

Non-labile organic P (NL-Po) represents the least active component of organic P, with low bioavailability [90]. NL-Po accumulates in soils due to the strong adsorption of negatively charged organic P to clays and hydrous Fe- and Al-oxides, facilitated by polyvalent bridging cations like Ca^2+^ or Fe^3+^. Additionally, under acidic conditions, DNA may penetrate the interlayer spaces of clays, becoming highly non-labile [91]. Soils that receive excessive P fertilization, mainly in the form of organic fertilizers, tend to accumulate non-labile organic P [92]. In the case of the rice-based cropping system, a statistically lower level of NL-Po was obtained compared to other cropping systems. This difference might be attributed to the relatively lower application of organic nutrient sources, influenced by various factors.

### Residual P fraction

Residual P is an unextractable recalcitrant-P fraction that forms strong bonds with soil particles, resulting in very low availability [93, 94]. This fraction comprises unextractable organic P or inert inorganic P (refractory P compounds), as well as a small amount of undetermined organic P within the extractable P fractions [95]. Among all the other fractions, the residual P fraction exhibits relatively high levels and can supply P for a longer period. In the vegetable-based, fruit-based, maize-based, and rice-based cropping systems, the residual P fraction accounts for 31.74%, 37.23%, 45.64%, and 47.19% of the total P, respectively. The moderate concentration of residual P could be attributed to the presence of moderately weathered soils. Typically, residual P exceeds 30% of the total P in moderately weathered soils, whereas it surpasses 80% in strongly weathered soils [96]. The significantly lower levels of residual P in the vegetable-based system compared to the maize-based and rice-based cropping systems could be attributed to the higher content of organic and inorganic pools during the sequential extraction process.

### Total P

Total P determines the overall P content in soils, encompassing all forms [97]. The relatively high total P content could be attributed to the presence of phosphorus-rich parent materials. Soils developed on granite gneiss typically exhibit the highest total P content, followed by shales with basic intrusion, limestone with intrusions of micaceous schist, and quartzite [98].

## Conclusions

The study conducted in the central mid-hill region of Nepal revealed the prevalence of vegetable-based, fruit-based, rice-based, and maize-based cropping systems in the area. These cropping systems exhibit variations in nutrient management, cropping intensity, and topography. The high variability observed in general soil parameters, including pH, organic matter content, cation exchange capacity, and proportions of sand, silt, and clay, can be attributed to differences in parent materials, weathering processes, and nutrient management practices. The vegetable-based system exhibited higher levels of most P fractions, followed by the fruit-based, maize-based, and rice-based systems. This indicates that the vegetable-based system can supply more phosphate ions for different periods (short, medium, and long) compared to other systems. In the study area, the residual P fraction contributed comparatively more for total P, suggesting that it may serve as a future stock P source for plant uptake. This study provides valuable insights into the diversity of cropping systems and their impact on P fractionations. Such knowledge can contribute to the development of improved soil P management strategies and promote sustainable agricultural practices in the region.
